# Determination of Chemical Irritation Potential Using a Defined Gene Signature Set on Tissue-Engineered Human Skin Equivalents

**DOI:** 10.1016/j.xjidi.2021.100011

**Published:** 2021-03-15

**Authors:** Amy L. Harding, Craig Murdoch, Simon Danby, Md Zobaer Hasan, Hirofumi Nakanishi, Tetsuo Furuno, Sirwan Hadad, Robert Turner, Helen E. Colley

**Affiliations:** 1 The School of Clinical Dentistry, The University of Sheffield, Sheffield, United Kingdom; 2 Sheffield Dermatology Research, Department of Infection, Immunity and Cardiovascular Disease, The Medical School, The University of Sheffield, Sheffield, United Kingdom; 3 Safety Design Centre, Rohto Pharmaceutical Co, Ltd, Kyoto, Japan; 4 Sheffield Teaching Hospitals NHS Foundation Trust, Sheffield, United Kingdom; 5 Research Software Engineering Sheffield, The University of Sheffield, Sheffield, United Kingdom

**Keywords:** CA, cinnamaldehyde, CAP, capsaicin, Co-DEA, cocamide diethanolamine, Co-MEA, cocamide monoethanolamine, CON, control, H_2_O, water, HDF, human dermal fibroblast, HSE, human skin equivalent, KC, keratinocyte, LA, lactic acid, LDA, linear discrimination analysis, LDH, lactate dehydrogenase, MMP, matrix metalloproteinase, MP, methylparaben, N-LA, neutralized lactic acid, PCA, principal component analysis, TEER, transepithelial electrical resistance

## Abstract

There are no physical or visual manifestations that define skin sensitivity or irritation; a subjective diagnosis is made on the basis of the evaluation of clinical presentations, including burning, prickling, erythema, and itching. Adverse skin reaction in response to topically applied products is common and can limit the use of dermatological or cosmetic products. The purpose of this study was to evaluate the use of human skin equivalents based on immortalized skin keratinocytes and evaluate the potential of a 22-gene panel in combination with multivariate analysis to discriminate between chemicals known to act as irritants and those that do not. Test compounds were applied topically to full-thickness human skin equivalent or human ex vivo skin and gene signatures determined for known irritants and nonirritants. Principle component analysis showed the discriminatory potential of the 22-gene panel. Linear discrimination analysis, performed to further refine the gene set for a more high-throughput analysis, identified a putative seven-gene panel (*IL-6, PTGS2, ATF3, TRPV3, MAP3K8, HMGB2*, and matrix metalloproteinase gene *MMP-3*) that could distinguish potential irritants from nonirritants. These data offer promise as an in vitro prediction tool, although analysis of a large chemical test set is required to further evaluate the system.

## Introduction

Skin sensitivity or irritation can be induced by exposure to exogenous stimuli that can be physical, in the form of UV light and wind; environmental, such as atmospheric pollutants; thermal, manifesting as heat or cold; or chemical entities, for example, constituents of cosmetics, H^+^ ions, and drugs (Talagas and Misery, 2019). The topical application of dermatological agents that cause adverse skin sensitivity or irritation is a common reason for poor treatment compliance and can restrict therapeutic options. Moreover, adverse skin reaction owing to cosmetics and skincare products is a significant problem affecting a large proportion of individuals, with 78% of people with sensitive skin reporting avoidance of dermatological products because of potential adverse effects (Farage et al., 2006). Currently, there are no physical or visual manifestations that define skin sensitivity, with 50% of adults reporting dermal sensitivity without any other clinical signs of inflammation (Ständer et al., 2009). Therefore, a subjective diagnosis is based on various sensory clinical manifestations, including burning, tingling, stinging, prickling, and itching (Misery et al., 2017). This range of symptoms is collectively termed sensitive skin syndrome and can affect people with seemingly healthy skin. Skin irritation is more closely related to inflammation that may be initiated in a specific or nonspecific manner. The molecular mechanism for both skin sensitivity and irritation is still poorly defined and is likely to consist of interplay between keratinocytes (KCs) and dermal fibroblasts, which constitute the main mass of cells in the skin, along with other cell types from the neuronal and immune lineage.

Numerous patient-based methods have been proposed to test for sensitization and irritancy, including corneometry, transepidermal water loss, quantitative sensory testing, and thermal sensation tests (Marriott et al., 2005). The lactic acid (LA) stinging test is also commonly used to determine the tolerability of sensitive skin to a given chemical (Frosch and Kligman, 1977). For many decades, alternative endpoints of skin sensitization and irritancy have utilized animal-based in vivo assays; however, none of these tests provide a clear standardized measurable outcome to predict skin reaction, and they are not compatible with high-throughput testing that is required by industry.

This, combined with European Union directives prohibiting the use of animal testing for cosmetics (2003/15/EU, 2010/63/EU, and European Union regulation 1223, 2009), has seen the development, acceptance, and rapid rise of nonanimal alternative in vitro assays for skin sensitization and irritancy. Such methods include protein-binding interactions using the direct peptide reactivity assay (Gerberick et al., 2004). Current cell-based tests include those using myeloid cancer cell lines, such as the human cell line activation test that measures changes in CD86 and CD54 expression in THP-1 monocytes (Ashikaga et al., 2006; Sakaguchi et al., 2006); U-SENS, based on a similar readout using U937 cells (Alépée et al., 2015; Piroird et al., 2015); and those based on monolayer-cultured KC reporter-based assay systems, including KeratinoSens (Andreas et al., 2011; Emter et al., 2010) and LuSens (Ramirez et al., 2014), or secretion of IL-18 (Corsini et al., 2013), some of which have been accepted by or are in review at the Organisation for Economic Co-operation and Development as approved standard tests. An in vitro version of the LA stinging test has also been developed (Sakka et al., 2018).

However, these tests are based on cells in monoculture, whereas skin is composed of a stratified squamous epithelium containing KCs displaying increasing levels of differentiation, with a granular layer and stratum corneum that play a significant role in skin permeability to topically applied compounds. The use of tissue-engineered human skin equivalents (HSEs) for both skin sensitivity and irritancy aims to overcome the deficiencies of simple monoculture assays.

Characterization of skin sensitivity and irritancy at the gene level is rapidly gaining pace and has driven the development of several gene expression-based assay systems using either reconstituted human epidermis skin equivalents that consist solely of a stratified squamous epidermis (Hasan et al., 2019; Saito et al., 2017) or full-thickness HSE (consisting of both epidermis and a fibroblast-populated dermis), with SENS-IS being the most advanced in terms of validation against a large panel of chemicals (Cottrez et al., 2016). We recently reported whole genome expression profiles of reconstituted human epidermis in response to LA, identifying several genes highly associated with irritancy and sensitization (Hasan et al., 2019). Here, we report the use of a full-thickness HSE system based on immortalized skin KCs and show that a 22-gene panel can discriminate between chemicals known to act as irritants from those that do not. Moreover, we use linear discrimination analysis (LDA) to further refine the gene set for more high-throughput analysis, narrowing the panel down to seven genes that correctly cluster irritants from nonirritants.

## Results

### Histological analysis of human skin and HSEs after treatment with chemical compounds

**Figure 1:**
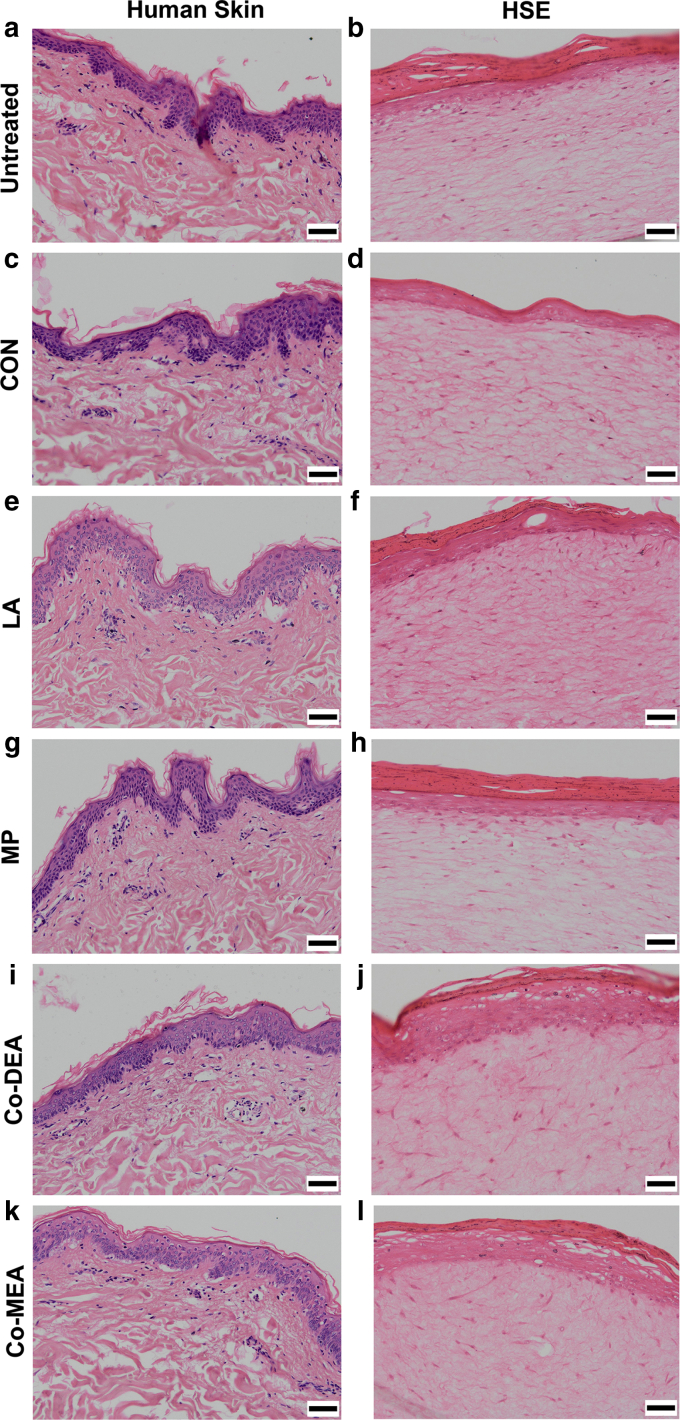
**Morphological characterization of human skin and HSEs after topical application of chemical compounds.** Representative images of H&E-stained sections of human skin (left panels) and HSE (right panels) after exposure to chemical compounds for 24 hours. (**a, b**) Untreated, (**c, d**) vehicle H_2_O CON, (**e, f**) LA (5%), (**g, h**) MP (0.2%), (**i, j**) Co-DEA (2%), and (**k, l**) Co-MEA (2%). Bar = 50 μm; n = 3, with skin from a different donor used in each independent experiment. Co-DEA, cocamide diethanolamine; Co-MEA, cocamide monoethanolamine; CON, control; H_2_O, water; HSE, human skin equivalent; LA, lactic acid; MP, methylparaben.

LA and other cosmetic compounds—methylparaben (MP), cocamide diethanolamine (Co-DEA), or cocamide monoethanolamine (Co-MEA)—were applied topically to ex vivo human skin and HSE for 24 hours. Histological analysis of the untreated skin and HSE revealed a full-thickness stratified squamous epithelium with signs of epithelial desquamation and a dermal fibroblast-populated dermis (Figure 1a and b), the structure of which was not altered on treatment with the vehicle control (CON), water (H_2_O) (Figure 1c and d). In contrast, LA caused marked disruption to the epidermis in both skin and HSE in comparison to CON, where extensive detachment of the epidermis from the basement membrane was observed in ex vivo skin but not in HSE. In contrast, epithelial vacuolation was observed in the stratum spinosum of HSE, but this was less obvious in ex vivo skin (Figure 1e and f). Treatment with MP caused no morphological effects in skin or HSE (Figure 1g and h). Ex vivo skin showed no histological changes on treatment of Co-DEA, whereas HSE displayed occasional vacuolation in the stratum spinosum (Figure 1i and j). Treatment of ex vivo skin with Co-MEA caused epidermal detachment of the basement membrane in places with occasional epithelial vacuolation, whereas for HSE, only vacuolation in the stratum spinosum was observed (Figure 1k and l).

### Epithelial integrity of human skin and HSE after incubation with chemical compounds

**Figure 2:**
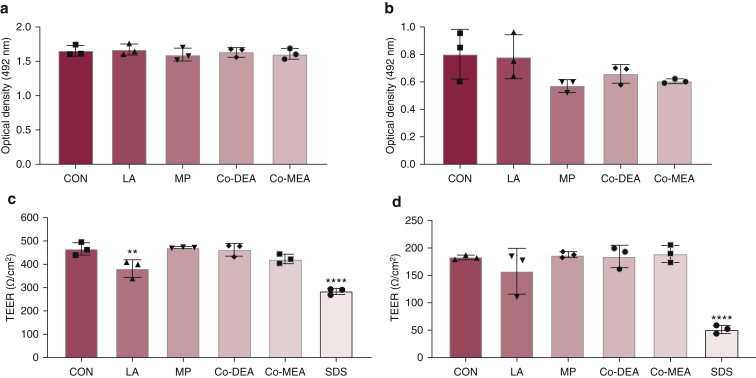
**Epithelial integrity after topical exposure to chemical compounds.** LDH release and TEER measurements for (**a, c**) human skin and (**b, d**) HSEs after exposure to compounds for 24 hours: LA (5%), MP (0.2%), Co-DEA (2%), Co-MEA (2%), and SDS (5%). Data presented as mean ± SD. ∗∗*P* <0.01 and ∗∗∗∗*P* <0.0001 as analyzed by ordinary one-way ANOVA with Dunnett’s multiple comparison compared with vehicle CON; n = 3, with skin from a different donor used in each independent experiment. Co-DEA, cocamide diethanolamine; Co-MEA, cocamide monoethanolamine; CON, control; HSE, human skin equivalent; LA, lactic acid; LDH, lactate dehydrogenase; MP, methylparaben; TEER, transepithelial electrical resistance.

In many instances, epithelial vacuolation in response to chemical stimuli is transient and reversible, and so its presence may not directly correlate with tissue damage (Shubin et al., 2016). Therefore, release of lactate dehydrogenase (LDH) by cells in the skin or HSE was used as a measure of cellular damage after topical exposure to the compounds for 24 hours. No significant differences in LDH release were observed for both skin and HSE after treatment with any of the chemical compounds (Figure 2a and b). Tissue integrity, measured using transepithelial electrical resistance (TEER), showed a similar trend with only a significant reduction observed after treatment with LA (*P* = 0.005) for human skin. Treatment with 5% SDS, a detergent known for its epithelial-disrupting properties, significantly reduced the TEER readings compared with all other treatments for both human skin and HSE (*P* < 0.0001) (Figure 2c and d).

### Gene expression profile of human skin and HSE in response to chemical compounds

**Figure 3:**
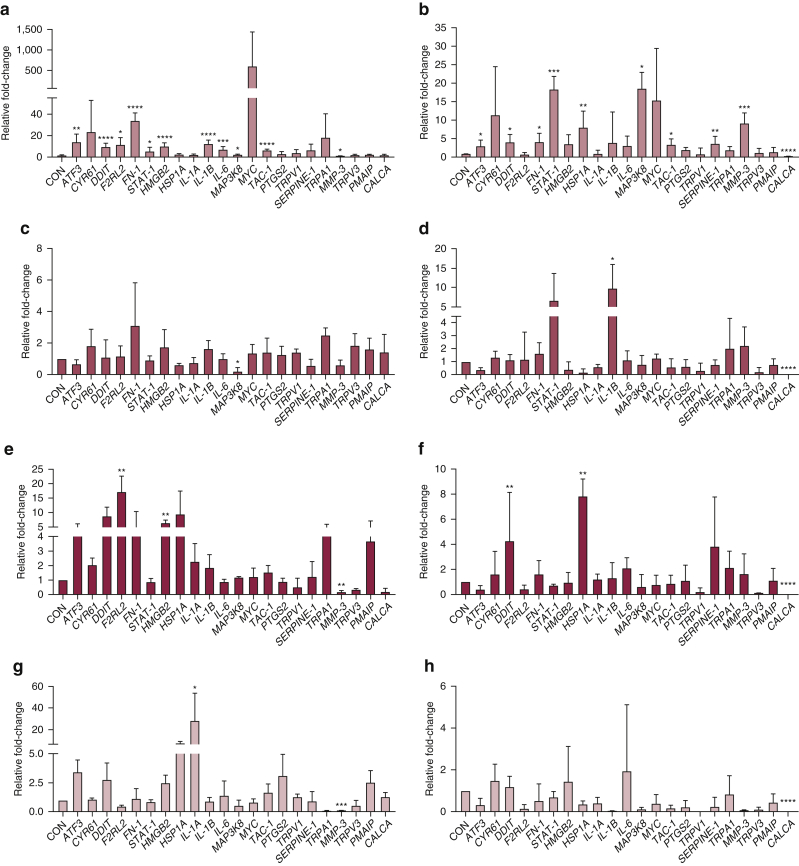
**RT-qPCR expression data for 22 genes of interest after treatment with chemical compounds.** Fold-change differences in gene expression for human skin (left panels) and HSE (right panels) in response to exposure to chemical compounds for 24 hours. (**a, b**) LA (5%), (**c, d**) MP (0.2%), (**e, f**) Co-DEA (2%), and (**g, h**) Co-MEA (2%). The Ct of each test gene was normalized against the *U6* reference gene, and then fold-changes in gene expression relative to the H_2_O-treated CON group were calculated using the formula 2^-ΔΔCt^. Data are presented as mean ± SD. ∗*P* =0.05, ∗∗*P* <0.01, ∗∗∗*P* <0.001 and ∗∗∗∗*P* <0.0001. Genes were analyzed by ordinary one-way ANOVA for each treatment with Dunnett’s multiple post-hoc comparison test compared with vehicle control; n = 3, with skin from a different donor used in each independent experiment. Co-DEA, cocamide diethanolamine; Co-MEA, cocamide monoethanolamine; CON, control; FN, fibronectin; H_2_O, water; HSE, human skin equivalent; HSP, heat shock protein; LA, lactic acid; MMP, matrix metalloproteinase; MP, methylparaben; STAT, signal transducer and activator of transcription; TAC, transit amplifying cell.

Gene expression analysis for a panel of 22 genes previously associated with a LA response (Hasan et al., 2019) was performed by qPCR and fold-changes in expression compared with vehicle-treated CONs (Figure 3). Treatment of skin with LA revealed a significant fold-increase in *AFT3* (*P* = 0.0027), *DDIT* (*P* < 0.0001), *F2RL2* (*P* = 0.0192), fibronectin gene *FN-1* (*P* < 0.0001), signal transducer and activator of transcription gene *STAT-1* (*P* = 0.0204), *HMGB2* (*P* < 0.0001), *IL1β* (*P* < 0.0001), *IL-6* (*P* = 0.0009), *MAP3K8* (*P* = 0.0133), and transit amplifying cell gene *TAC-1* (*P* < 0.0001), and a fold-decrease in expression observed for matrix metalloproteinase (MMP) gene *MMP-3* (*P* = 0.0364) compared with CON (Figure 3a). A similar cohort of genes were also affected when HSEs were treated with LA with a significant fold-increase observed for *ATF3* (*P* = 0.0235), *DDIT* (*P* = 0.0214), fibronectin gene *FN-1* (*P* = 0.0270), signal transducer and activator of transcription gene *STAT-1* (*P* = 0.0004), heat shock protein gene *HSP1A* (*P* = 0.0050), *MAP3K8* (*P* = 0.0151), transit amplifying cell gene *TAC-1* (*P* = 0.0143) and *SERPINE1* (*P* = 0.0091). In contrast to skin, *MMP-3* (*P* = 0.0003) expression was significantly increased and *CALCA* (*P* < 0.0001) was significantly decreased in HSEs (Figure 3b). Exact fold-changes in gene expression for skin and HSE are provided in Tables 1 and 2.

**Table 1:** RT-qPCR Expression Data for 22 Genes of Interest after Exposure of Human Skin with Chemical Compounds

Gene	LA	MP	Co-DEA	Co-MEA
*ATF3*	13.93 ± 7.29^1^	0.67 ± 0.27	4.77 ± 1.39	3.43 ± 1.01
*CYR61*	23.53 ± 29.24	1.81 ± 1.06	2.05 ± 0.47	1.05 ± 0.10
*DDIT*	9.49 ± 3.13^1^	1.11 ± 1.09	0.86 ± 0.23	0.85 ± 0.16
*F2RL2*	11.61 ± 6.33^1^	1.18 ± 0.65	17.19 ± 5.42^1^	0.46 ± 0.09
*FN-1*	33.94 ± 7.19^1^	3.12 ± 2.72	4.25 ± 6.05	1.13 ± 0.85
*STAT-1*	5.43 ± 3.51^1^	0.91 ± 0.28	0.86 ± 0.23	0.85 ± 0.16
*HMGB2*	10.18 ± 2.70^1^	1.76 ± 1.10	6.37 ± 1.03^1^	2.46 ± 0.70
*HSP1A*	2.55 ± 0.42	0.61 ± 0.10	9.32 ± 8.15	6.97 ± 1.70
*IL-1A*	1.85 ± 0.83	0.75 ± 0.32	2.29 ± 1.22	28.11 ± 25.35^1^
*IL-1B*	12.21 ± 3.49^1^	1.65 ± 0.52	1.83 ± 0.92	0.88 ± 0.37
*IL-6*	6.96 ± 2.67^1^	1.00 ± 0.32	0.86 ± 0.18	1.41 ± 1.24
*MAP3K8*	1.86 ± 0.34^1^	0.21 ± 0.23^2^	1.18 ± 0.07	0.51 ± 0.47
*MYC*	598.60 ± 838.8	1.36 ± 0.54	1.2 ± 0.63	0.82 ± 0.30
*TAC-1*	6.18 ± 0.89^1^	1.44 ± 0.88	1.54 ± 0.46	1.69 ± 0.72
*PTGS2*	2.91 ± 1.84	1.27 ± 0.54	0.89 ± 0.23	3.1 ± 1.84
*TRPV1*	3.78 ± 2.97	1.41 ± 0.19	0.51 ± 0.62	1.29 ± 0.24
*SERPINE-1*	6.48 ± 5.13	0.58 ± 0.39	1.23 ± 1.04	0.92 ± 0.81
*TRPA1*	18.49 ± 21.49	2.51 ± 0.45	4.49 ± 1.50	0.03 ± 0.02
*MMP-3*	0.45 ± 0.34^2^	0.59 ± 0.32	0.16 ± 0.13^2^	0.02 ± 0.03^2^
*TRPV3*	1.65 ± 0.90	1.84 ± 0.75	0.35 ± 0.05	0.53 ± 0.44
*PMAIP*	2.08 ± 0.36	1.63 ± 0.68	3.67 ± 3.43	2.54 ± 1.00
*CALCA*	1.39 ± 1.22	1.43 ± 1.13	0.18 ± 0.24	1.28 ± 0.38

Abbreviations: Co-DEA, cocamide diethanolamine; Co-MEA, cocamide monoethanolamine; CON, control; FN, fibronectin; HSP, heat shock protein; LA, lactic acid; MMP, matrix metalloproteinase; MP, methylparaben; STAT, signal transducer and activator of transcription; TAC, transit amplifying cell.

Gene expression fold-change for human skin in response to exposure to chemical compounds for 24 hours—LA (5%), MP (0.2%), Co-DEA (2%), and Co-MEA (2%)—compared with relative CON samples. Data are expressed as fold-change relative to housekeeping gene *U6*. Data presented as mean ± SD; n = 3 from three different donors.

1 Genes with statistically significant fold-increase.

2 Genes with statistically significant fold-decrease.

**Table 2:** RT-qPCR Expression Data for 22 Genes of Interest after Exposure of HSEs with Chemical Compounds

Gene	LA	CAP	CA	N-LA	MP	Co-DEA	Co-MEA
*ATF3*	3.06 ± 1.63^1^	8.88 ± 2.11^1^	4.09 ± 1.14^1^	0.89 ± 0.05	0.37 ± 0.17	0.40 ± 0.29	0.33 ± 0.29
*CYR61*	11.47 ± 13.06	2.76 ± 0.83^1^	1.77 ± 0.95	0.15 ± 0.10	1.34 ± 0.46	1.63 ± 1.81	1.48 ± 0.77
*DDIT*	4.07 ± 2.13^1^	8.67 ± 3.96^1^	7.42 ± 2.18^1^	5.02 ± 1.01	1.13 ± 0.43	4.69 ± 1.07^1^	1.19 ± 0.49
*F2RL2*	0.82 ± 0.49	1.58 ± 0.38	0.93 ± 0.20	0.37 ± 0.46	1.80 ± 2.05	0.40 ± 0.34	0.15 ± 0.17
*FN-1*	4.27 ± 2.24^1^	26.87 ± 8.31^1^	50.90 ± 7.74^1^	18.12 ± 5.65^1^	1.66 ± 0.80	1.59 ± 1.11	0.52 ± 0.80
*STAT-1*	18.40 ± 3.50^1^	6.19 ± 2.32^1^	4.98 ± 1.20	4.67 ± 2.38	6.78 ± 6.89	0.73 ± 0.10	0.67 ± 0.27
*HMGB2*	3.65 ± 2.46	1.83 ± 0.30	1.99 ± 0.90	1.12 ± 0.16	0.39 ± 0.60	0.96 ± 0.79	1.46 ± 1.67
*HSP1A*	8.12 ± 4.28^1^	2,160,476 ± 3,741,437	158.8 ± 65.46	120.60 ± 81.85	0.17 ± 0.26	7.83 ± 1.37^1^	0.36± 0.14
*IL-1A*	1.02 ± 0.91	0.92 ± 0.16	0.61 ± 0.51	1.59 ± 0.34	0.59 ± 0.18	1.22 ± 0.39	0.40 ± 0.30
*IL-1B*	4.03 ± 3.4	0.16 ± 0.07^2^	0.99 ± 0.35	1.20 ± 0.30	9.79 ± 6.15^1^	1.32 ± 1.22	0.04 ± 0.02
*IL-6*	3.22 ± 2.50	0.72 ± 0.92	0.61 ± 0.26	0.70 ± 0.61	1.13 ± 0.70	2.12 ± 0.80	1.94 ± 3.16
*MAP3K8*	3.23 ± 1.22^1^	0.66 ± 0.36	3.35 ± 1.69^1^	0.62 ± 0.23	0.80 ± 0.69	0.65 ± 0.94	0.11 ± 0.09
*MYC*	15.42 ± 13.84	7.29 ± 2.44^1^	5.31 ± 3.00	1.88 ± 1.01	1.28 ± 0.30	0.80 ± 0.73	0.39 ± 0.43
*TAC-1*	3.39 ± 1.51^1^	21.99 ± 10.75^1^	4.45 ± 0.76	0.87 ± 0.12	0.57 ± 0.65	0.87 ± 0.68	0.16 ± 0.14
*PTGS2*	2.06 ± 0.56	3.73 ±1.51	2.42 ± 0.76	0.72 ± 0.62	0.62 ± 0.54	1.13 ± 1.18	0.23± 0.30
*TRPV1*	0.89 ± 1.54	0.37 ± 0.65	1.62 ± 0.45	2.84 ± 1.38	0.32 ± 0.56	0.193 ± 0.33	0.0001 ± 2.309e-005
*SERPINE-1*	3.83 ± 1.88^1^	3.74 ± 2.06^1^	2.02 ± 0.96	1.39 ± 0.46	0.78 ± 0.36	1.47 ± 0.16	0.25 ± 0.42
*TRPA1*	1.93 ± 0.93	2.97 ± 2.51	2.28 ± 0.57	1.03 ± 0.05	2.03 ± 2.30	2.14 ± 1.30	0.84 ± 0.88
*MMP-3*	9.19 ± 2.82^1^	0.95 ± 0.85	0.74 ± 0.68	0.18 ± 0.30	2.23 ± 1.46	1.63 ± 1.60	0.05 ± 0.03
*TRPV3*	1.24 ± 1.10	2.86 ± 0.99^1^	1.91 ± 0.17	0.31 ± 0.44	0.20 ± 0.35	0.09 ± 0.04	0.08 ± 0.13
*PMAIP*	1.46 ± 1.21	2.34 ± 0.46	2.45 ± 0.86	1.10 ± 0.77	0.78 ± 0.43	1.14 ± 0.94	0.45 ± 0.41
*CALCA*	0.029 ± 0.05^2^	9.34 ± 10.24	5.22 ± 0.58	2.44 ± 1.58	0.002 ± 0.004^2^	0.002 ± 0.004^2^	0.003 ± 0.005^2^

Abbreviations: CA, cinnamaldehyde; CAP, capsaicin; Co-DEA, cocamide diethanolamine; Co-MEA, cocamide monoethanolamine; CON, control; FN, fibronectin; HSE, human skin equivalent; HSP, heat shock protein; LA, lactic acid; MMP, matrix metalloproteinase; MP, methylparaben; N-LA, neutralized lactic acid; STAT, signal transducer and activator of transcription; TAC, transit amplifying cell.

Gene expression fold-change in response to exposure to chemical compounds for 24 hours— LA (5%), MP (0.2%), Co-DEA (2%), Co-MEA (2%), N-LA, CA (3%), and CAP (0.1%)—compared with relative CON samples. Data are expressed as fold-change relative to housekeeping gene *U6*. Data presented as mean ± SD. Genes were analyzed by ordinary one-way ANOVA for each treatment with Dunnett’s multiple comparison compared with vehicle control; n = 3.

1 Genes with statistically significant fold-increase.

2 Genes with statistically significant fold-decrease.

Treatment of skin and HSE with MP revealed few changes, with a significant fold-decrease in *MAP3K8* (*P* = 0.0224) for skin (Figure 3c) and increased expression of *IL1β* (*P* = 0.0225) and decreased expression of *CALCA* (*P* < 0.0001) for HSE compared with CON (Figure 3d). Treatment of skin with Co-DEA stimulated increased expression of *F2RL2* (*P* = 0.0012) and *HMGB2* (*P* = 0.0031) with fold-decreases observed for *MMP-3* (*P* = 0.0031) (Figure 3e) in skin, whereas Co-DEA increased expression of *DDIT* (*P* = 0.0072) and heat shock protein gene *HSP1A* (*P* = 0.0065) in HSE and decreased expression of *CALCA* (*P* < 0.0001) for HSE compared with CON (Figure 3f). Treatment of tissues with Co-MEA caused the fewest changes in gene expression, with significant increased expression of *IL1α* (*P* = 0.0474) and again a fold-decrease in *MMP-3* (*P* = 0.0010) for skin (Figure 3g) and only decreased expression of *CALCA* (*P* < 0.0001) for HSE compared with CON (Figure 3h).

### Multivariate analysis of chemical compound gene signatures in human skin and HSE

**Figure 4:**
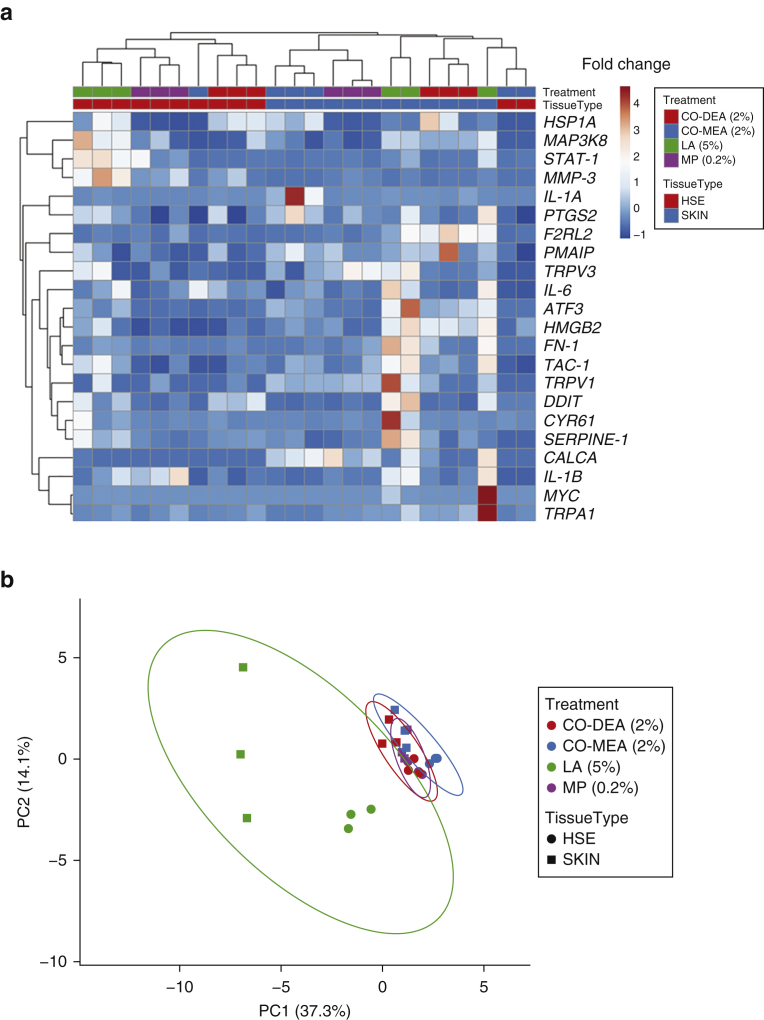
**Multivariate analysis of chemical stimulation on gene signatures in skin and HSEs.** Unsupervised hierarchical clustering of gene expression data for 22 genes after treatment with chemicals. In the heatmap visualization, each gene is represented by a single row and each chemical, a single column. (**a**) Red indicates increased fold-change in gene expression, whereas dark blue indicates decreased fold-change gene expression. Unsupervised hierarchical cluster analysis was performed using correlation distance and average linkage. (**b**) PCA representing gene expression profiles for skin (square) and HSE (circle). The score plot displaying PC1 and PC2 explains 37.3% and 14.1% of the total variance, respectively, after exposure. Prediction ellipses have a probability of 0.95 that a new observation from the same group will fall within the ellipse. Single-value decomposition with imputation was used to calculate the PC. Co-DEA, cocamide diethanolamine; Co-MEA, cocamide monoethanolamine; FN, fibronectin; HSE, human skin equivalent; HSP, heat shock protein; LA, lactic acid; MMP, matrix metalloproteinase; MP, methylparaben; PC, principal component; PCA, principal component analysis; STAT, signal transducer and activator of transcription; TAC, transit amplifying cell.

Hierarchical gene cluster analysis of the 22-gene panel showed that gene expression for skin and HSE clustered separately and distinguishable from each other, except for two of the three Co-DEA–treated HSE samples that clustered with skin. All other treatments displayed gene profiles that were distinguishable from each other based on hierarchical analysis; the exception was for LA-treated skin where one of the samples displaying high levels of *MYC* and *TRPA1* was unclustered (Figure 4a). Principal component analysis (PCA) showed that LA-treated skin and HSE clustered away from the other chemical compounds, indicating that the 22-gene panel differentiates between irritant and nonirritant compounds (Figure 4b). The nonirritants Co-DEA, Co-MEA, and MP clustered tightly together with overlapping prediction ellipses. In contrast, LA-treated skin and HSE clustered separately, suggesting that LA activates similar but slightly distinct gene responses in skin compared with HSE (Figure 4b). Based on these results, HSEs were used in all subsequent experiments.

### Differences in HSE gene expression profiles discriminating sensitizing compounds

**Figure 5:**
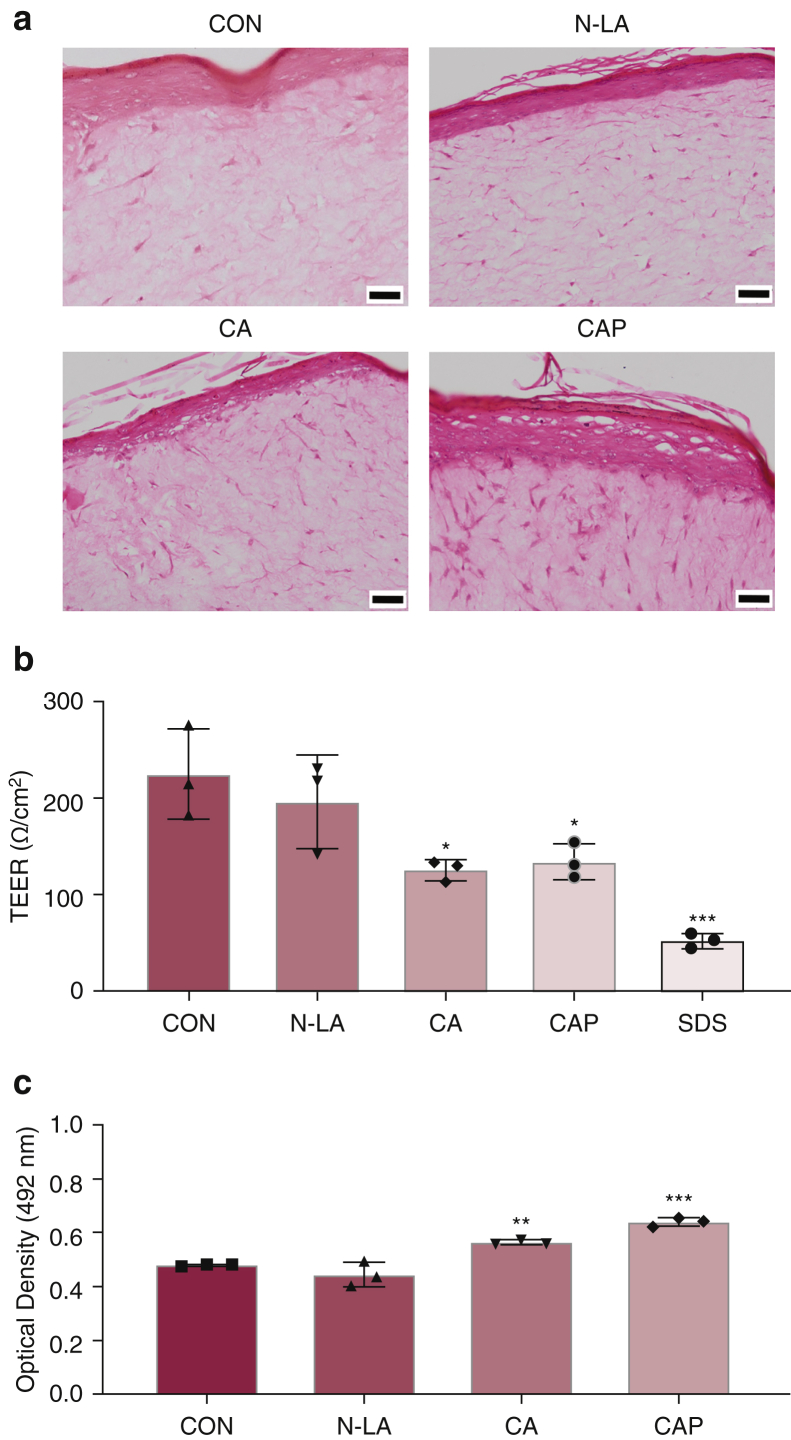
**Epithelial integrity of HSEs after topical exposure to chemical compounds.** (**a**) Representative images of H&E-stained sections of HSEs after exposure to chemical compounds for 24 hours: N-LA, CAP (0.1%), and CA (3%). Bar = 50 μm. (**b**) LDH release and (**c**) TEER measurements for HSEs after exposure to compounds for 24 hours: N-LA, CA (3%), CAP (0.1%), and SDS (5%). Data presented as mean ± SD. ∗*P* <0.05, ∗∗*P* <0.001 and ∗∗∗*P* <0.001 as analyzed by ordinary one-way ANOVA with Dunnett’s multiple comparison compared with vehicle CON; n = 3. CA, cinnamaldehyde; CAP, capsaicin; CON, control; HSE, human skin equivalent; LDH, lactate dehydrogenase; N-LA, neutralized lactic acid; TEER, transepithelial electrical resistance.

**Figure 6:**
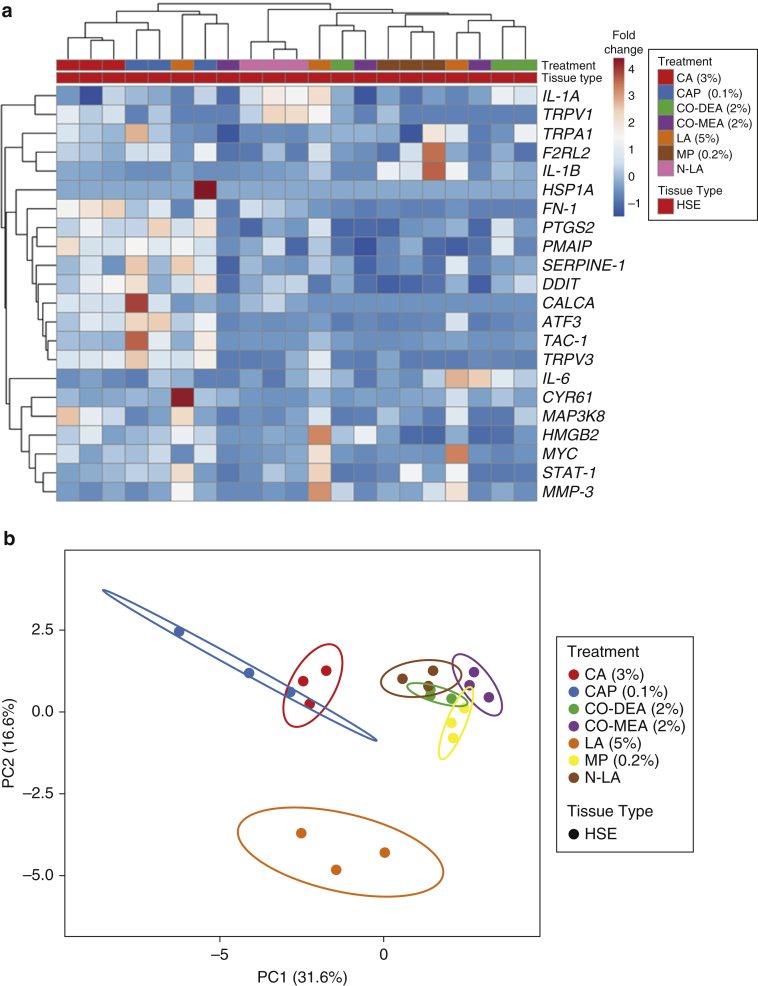
**Multivariate analysis of HSEs treated with known irritants and nonirritants.** (**a**) Hierarchical clustering of gene expression data for 22 genes after treatment with chemicals with heatmap visualization. Each gene is represented by a single row, and each chemical compound treatment is represented by a single column. Red indicates increased fold-change in gene expression, whereas dark blue indicates decreased fold-change gene expression. Hierarchical cluster analysis was performed using correlation distance and average linkage. (**b**) PCA representing gene expression profiles for HSE. The score plot displays PC1 and PC2 that explains 31.6% and 16.6% of the total variance, respectively, after exposure to chemicals. Prediction ellipses included with a probability of 0.95 that a new observation from the same group will fall inside the ellipse. Single-value decomposition with imputation was used to calculate PCs. CA, cinnamaldehyde; CAP, capsaicin; Co-DEA, cocamide diethanolamine; Co-MEA, cocamide monoethanolamine; FN, fibronectin; HSE, human skin equivalent; HSP, heat shock protein; LA, lactic acid; MMP, matrix metalloproteinase; MP, methylparaben; N-LA, neutralized lactic acid; PC, principal component; PCA, principal component analysis; STAT, signal transducer and activator of transcription; TAC, transit amplifying cell.

To further explore if the 22-gene panel can be used to discriminate irritant compounds, we expanded the test chemical set to include cinnamaldehyde (CA) and capsaicin (CAP) (classical irritants) and neutralized LA (N-LA) (a nonirritant). Histological analysis revealed signs of epithelial vacuolation in the basal epithelium of CA-treated HSE and within the stratum spinosum of CAP-treated HSE that was absent in both N-LA–treated and CON HSE (Figure 5a). Indeed, in this instance, both CA and CAP induced significant (*P* < 0.01) cytotoxicity and decreased tissue integrity (*P* ≤ 0.01) compared with treatment with vehicle CON when assessed by LDH release and TEER analysis, respectively, whereas treatment with N-LA had no effect (Figure 5b and c). Hierarchical cluster analysis of the gene panel showed that chemical irritants generally clustered together, with CA and CAP being closely associated, displaying increased gene expression in a number of common genes (Figure 6a and Table 2). PCA of gene expression profiles exemplified this with Co-DEA, Co-MEA, MP, and N-LA displaying overlapping prediction ellipses, whereas LA-treated HSEs present as a separate cluster (Figure 6b), suggesting that not only can the 22-gene panel distinguish irritant from nonirritant compounds but also that it has the potential to differentiate between different chemical classes of irritant compounds.

### LDA

**Figure 7:**
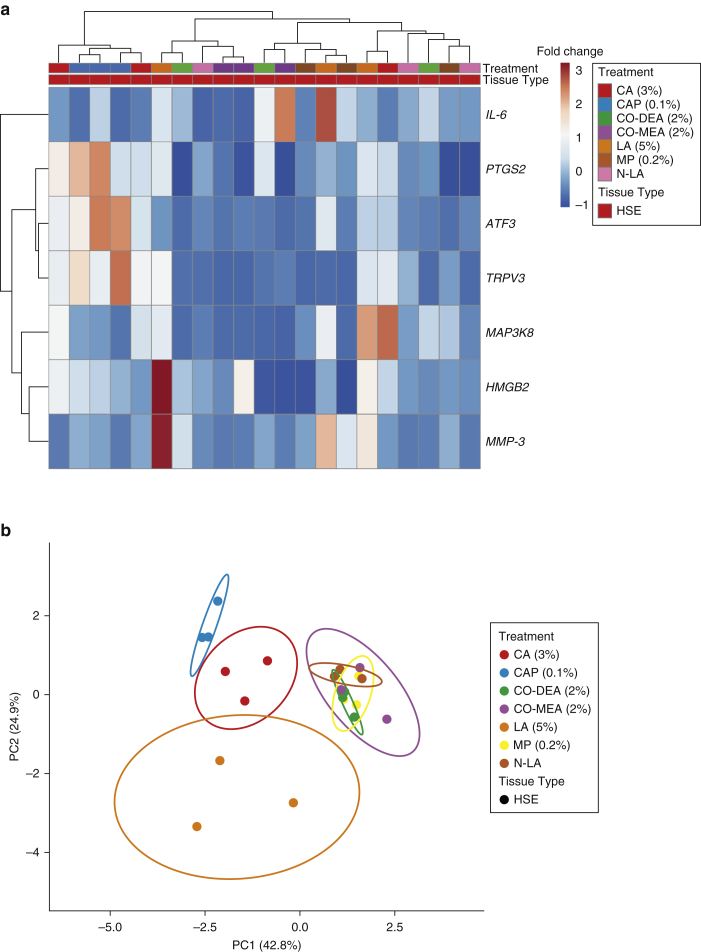
**Multivariate analysis and machine learning approach to identify irritant gene signature in HSEs.** (**a**) LDA was performed and identified seven genes with coefficients above 0.5 (*IL-6*, *PTGS2*, *ATF3*, *TRPV3*, *MAP3K8*, *HMGB2*, and *MMP-3*). Hierarchical clustering of gene expression data for the seven genes of interest after treatment with chemical compounds is shown with heatmap visualization. Each gene is represented by a single row, and each chemical compound treatment is represented by a single column. Red indicates increased fold-change in gene expression, whereas dark blue indicates decreased fold-change gene expression. (**b**) PCA plot representing gene expression profiles for HSE. PC1 indicates 42.8% and PC2 indicates 24.9% of the total variance after exposure to chemical compounds. Prediction ellipses included with a probability of 0.95 that a new observation from the same group will fall inside the ellipse. Single-value decomposition with imputation was used to calculate PCs. CA, cinnamaldehyde; CAP, capsaicin; Co-DEA, cocamide diethanolamine; Co-MEA, cocamide monoethanolamine; HSE, human skin equivalent; LA, lactic acid; LDA, linear discrimination analysis; MMP, matrix metalloproteinase; MP, methylparaben; N-LA, neutralized lactic acid; PC, principal component; PCA, principal component analysis.

A 22-gene set is a relatively large panel to routinely examine the potential of any given compound to induce irritation. It is possible that a number of genes in the panel are redundant or contribute little to the overall cluster analysis and that a smaller panel could be used with similar success. We used LDA to interrogate the gene panel to define a reduced cohort of genes with maintained effectiveness at identifying irritation potential. LDA identified seven genes with coefficients above 0.5 (*IL-6, PTGS2, ATF3, TRPV3, MAP3K8, HMGB2*, and *MMP-3*) (Table 3) that when reanalyzed for their hierarchical clustering profiles (Figure 7a) and PCA (Figure 7b), retained their ability to discriminate irritant from nonirritant chemicals.

**Table 3:** Linear Discriminant Coefficients for the 22-Gene Panel

Gene	LDA Coefficient
*ATF3*	1.009
*CYR61*	0.364
*DDIT*	0.169
*F2RL2*	−0.09
*FN-1*	0.288
*STAT-1*	0.178
*HMGB2*	0.610
*HSP1A*	3.0E-07
*IL-1A*	0.066
*IL-1B*	0.157
*IL-6*	1.055
*MAP3K8*	2.620
*MYC*	0.006
*TAC-1*	0.364
*PTGS2*	0.527
*TRPV1*	−1.459
*SERPINE-1*	−2.104
*TRPA1*	−0.332
*MMP-3*	1.435
*TRPV3*	0.772
*PMAIP*	0.359
*CALCA*	0.495

Abbreviations: FN, fibronectin; HSP, heat shock protein; LDA, linear discrimination analysis; MMP, matrix metalloproteinase; STAT, signal transducer and activator of transcription; TAC, transit amplifying cell.

## Discussion

Skin irritation or sensitivity has been attributed to the release of mediators that may instigate inflammation, disruption of the stratum corneum, and/or induction of neuronal hypersensitivity (Misery et al., 2017). Consumers of dermatological products require adequate protection against chemicals that have the potential to cause adverse skin reaction, and this, combined with legislative changes, has encouraged researchers to develop more robust and standardized in vitro skin assays. Using microarray technology, our previous work on reconstituted HSE identified a 25-gene panel that was associated with an LA-induced skin reaction (Hasan et al., 2019). Reconstituted HSEs are comprised solely of skin KCs and so lack the paracrine interplay with dermal fibroblasts that also contributes to a skin reaction. Therefore, in this study, we used full-thickness HSE as a more representative model. Studies with similar experimental setups using normal human skin KCs have found significant donor-to-donor variability, with disparity in HSE responses dependent on the batch of KCs used (Cottrez et al., 2016). To minimize this experimental variation, we have used telomerase reverse transcriptase (TERT)-immortalized skin KCs to reduce genetic background and to assist in assay transferability, an important consideration if the technology is to be adopted by other laboratories.

As a proof-of-principle investigation, we initially examined the response of HSE to 5% LA, a known irritant that induces an inflammatory and stress-response pathway (Hasan et al., 2019; Rendl et al., 2001). We also tested the chemical preservative MP and Co-DEA and Co-MEA, surfactants present in many cosmetic products, and compared these with freshly excised human skin treated with the same chemicals. We chose concentrations of MP, Co-DEA, and Co-MEA that are known to be nonirritant so that differences between irritant and nonirritant agents could be assessed.

Although histological examination showed signs of epithelial vacuolation in some treated samples, LDH and TEER analysis suggested that this was not translated to epithelial cell damage. Hierarchical clustering of gene expression data followed by PCA revealed that this 22-gene panel could effectively discriminate between LA and the nonirritants MP, Co-DEA, and Co-MEA. The nonirritants clustered closely together for both HSE and ex vivo skin, whereas gene responses to LA clustered into two distinct clusters, one for HSE and one for skin. This is likely because of LA interacting with a more extensive repertoire of cell types that are present within ex vivo skin (vascular, nerve, immune cell) as compared with HSE that are composed of just KCs and fibroblasts. For example, upregulation of genes for *TRPA1* and *TRPV1*, which have previously been associated with sensory neuron activation in the epidermis (Nielsen et al., 2018), was observed in ex vivo skin but not in HSE. Nevertheless, HSE composed of KCs and fibroblasts was still proficient at discriminating between the two classes of compounds, suggesting that this simplified in vitro skin model still has the power to discriminate between irritant and nonirritant chemicals. Addition of N-LA to HSEs reversed the gene expression profile from an irritant to a nonirritant phenotype that clustered with MP, Co-DEA, and Co-MEA by PCA, reinforcing the specificity of the gene panel and the ability of HSE to detect and respond to changes in H^+^ ions. To underline this, stimulation of HSE with CA and CAP induced epithelial damage and clustered as irritants but with a distinct gene profile to LA, reflecting the ability of these molecules to signal via different pathways.

Although made up of entirely different genes apart from *MMP-3* and *IL1α*, the discriminatory power of our 22-gene signature panel reflects closely to that of the 38 REDOX and inflammatory gene panel used in the SENS-IS assay (Cottrez et al., 2016). This is quite remarkable given the fact that our 22-gene set was derived using an unbiased microarray experimental approach (Hasan et al., 2019), whereas the SENS-IS 38-gene panel was chosen from an initial 900 gene set identified from data mining and review of the published literature from in vivo data from murine and human studies (Cottrez et al., 2015). The SENS-IS assay has been rigorously tested against more than 150 chemical compounds, positively identifying irritants based on a readout of any seven positively upregulated genes from the 38-gene panel, with high specificity, sensitivity, and accuracy reported (Cottrez et al., 2016). However, the use of large gene signature sets is cumbersome, and they do not lend themselves to easy analysis and high-throughput testing. Various integrated machine learning and bioinformatics approaches have been used to improve the prediction of toxicology and adverse reaction assays (Del Bufalo et al., 2018; Patlewicz et al., 2014). Here, we used LDA to refine our panel to identify the genes that contribute most to define irritation and found that a panel of just seven genes (*ATF3, MAP3K8, IL-6, PTGS2, TRPV3, HMGB2,* and *MMP-3*) were still discriminatory based on PCA. These genes cover a range of pathways, including inflammation, cell viability, damage, and extracellular remodeling, and have been identified in previous reports examining skin irritation or sensitivity.

ATF3 is a member of the CREB family of transcription factors whose expression is induced in response to cell stress, and on activation, ATF3 promotes gene transcription of factors that regulate metabolism, apoptosis, and inflammation (Thompson et al., 2009). Previous studies have shown increased *ATF3* gene expression in monolayer cultures of skin KCs in response to chemical insult and UVR (Schaper-Gerhardt et al., 2018), and increased protein levels have been observed in the epidermis of patients with the inflammatory skin disease erythema multiforme (Pollack et al., 2010). Moreover, *ATF3* was identified as a key upregulated gene in an in vitro reconstituted epidermis-only HSE in response to a number of skin irritants (Saito et al., 2013) and in gene dysregulation network analyses for genes implicated in toxicity and sensitization (Pronk et al., 2013), implicating this transcription factor in regulating skin immune responses to irritants. Likewise, MAP3K8 is a key regulator of the innate immune response (Arthur and Ley, 2013) and is responsible for activation of extracellular signal–regulated kinase 1/2 and p38 MAPK, whose expression have been implicated in skin inflammation in response to irritants in in vivo experimental models (Pastore et al., 2005) and in reconstituted HSE (Frankart et al., 2012). In addition, on cell stress, MAPK increases the expression of *ATF3* in skin KCs (Harper et al., 2005), thereby linking these two transcription factors.

Along with these two inflammatory-associated transcription factors, gene expression of the inflammatory mediators IL-6 and PTGS2 (often termed cyclooxygenase-2) was also identified. Increased gene expression of *IL-6* was detected in human skin in response to the irritant nonanoic acid using microarray analysis (Clemmensen et al., 2010). Elevated *IL-6* expression has also been detected in two-dimensional monolayers of cultured primary skin KCs, HaCaT cells, and dermal fibroblasts (Jung et al., 2016; Terunuma et al., 2001). Tsai et al. (2016) detected elevated secretion of IL-6 using a full-thickness HSE constructed of HaCaT KCs in response to a number of skin irritants, and Schmalz et al. (1998) observed similar findings when HSEs constructed of primary cells were incubated with metal-containing ceramics, whereas Bock et al. (2018) found that IL-6 secretion was only increased when MUTZ-3 Langerhans-like cells were incorporated into their HSE on stimulation with irritants. IL-6 was also dramatically increased in a dermal fibroblast only–populated three-dimensional model in response to cadmium chloride and lauryl sulfate (Augustin and Damour, 1995), suggesting that fibroblasts may be an important source of IL-6 in skin.

PTGS2 gives rise to PGs, molecules that are also involved in inflammation and can promote KC proliferation, aiding wound healing (Sato et al., 1997). Increased *PTGS2* gene expression was previously detected in human skin samples exposed to the known irritants lauryl sulfate and nanonic acid (Clemmensen et al., 2010). Increased *PTGS2* expression has also been observed in the suprabasal epidermal region of guinea pig skin in response to iodide (Nyska et al., 2001) and in murine epidermis in retinol- or benzalkonium chloride–induced dermatitis (Lee et al., 2010). Likewise, using a full-thickness HSE, Black et al. (2010a, 2010b) detected increased expression of PTGS2, also in the suprabasal region, in response to the vesicant 2-chloroethyl ethyl sulfide, and this appears to be mediated by activation of MAPK. Dermal fibroblasts were also found to secrete PGs in a PTGS2-dependent manner in both a three-dimensional fibroblast-containing collagen matrix and a murine model of irritant contact dermatitis (Saalbach et al., 2015; Sato et al., 1997). Taken together, these data provide strong evidence for the role of inflammatory mediates in the response to irritants and their usefulness in predictive irritant screening.

Our seven-gene panel also includes TRPV3, a receptor expressed by epidermal KCs with barrier function properties (Cheng et al., 2010; Peier et al., 2002). Several lines of evidence link TRPV3 with skin irritation. A number of plant-derived molecules containing known irritants, such as carvacrol, eugenol, and thymol, have been shown to activate KC-expressed TRPV3 (Xu et al., 2006). Moreover, activation of TRPV3 by α-hydroxyl acid–containing compounds causes excessive KC exfoliation that is linked with skin irritation (Cao et al., 2012), and ablation of *TRPV3* attenuated skin lesions in mice (Qu et al., 2019).

HMGB2 is a DNA-binding protein that facilitates the activity of transcription factors, although it can be released from necrotic cells where it has been shown to have a role in inflammation (Taniguchi et al., 2018). We previously showed that *HMGB2* is upregulated in reconstituted HSE when subjected to LA (Hasan et al., 2019). Although there are very few studies that examine its role in the skin, evidence from cisplatin- and benzopyrene-induced skin cytotoxicity experiments suggest that HMGB2 released from necrotic KCs triggers an immune response (Sharma et al., 2008), suggesting that a similar mechanism may occur in response to skin irritants.

MMP-3 (or stromelysin-1) is a protease with a broad specificity for many connective tissue extracellular matrix proteins and has been implicated in playing a role in skin pathology. For instance, MMP-3–deficient mice displayed an impaired response to topical treatment with the potent irritant dinitrofluorobenzene (Wang et al., 1999). Clemmensen et al. (2010) observed epidermal expression of MMP-3 in experimental irritant contact dermatitis in human skin and suggested that secretion of this protease may release GFs that are sequestered in the extracellular matrix as part of a tissue repair process. The gene transcript for *MMP-3* has been detected previously by us and others on analysis of either reconstituted human epidermis or full-thickness HSE when these models were treated with several irritants (Cottrez et al., 2016, 2015; Hasan et al., 2019; Petry et al., 2018), underscoring its significance as a marker of skin irritation.

Our data provide good evidence that HSEs based on KCs and fibroblasts alone have the ability to discriminate between irritant and nonirritant chemicals. However, skin is composed of many different cell types with neuronal and immune components, in particular, known to markedly influence skin irritation and sensitivity reactions. Indeed, the presence of neuronal and immune cells in ex vivo skin is the likely reason for the difference we observed in gene expression profiles of skin and HSE toward LA. Although the HSE used in this study has discriminatory power, the lack of a neuronal and immune component highlights its limitations. The move toward advanced HSE is well underway, with published reports of innervated skin equivalents, although these in vitro models have used rodent sensory neurons in combination with human cells (Blais et al., 2014; Cadau et al., 2015; Lebonvallet et al., 2012), which raises concerns about cross-species paracrine signaling and the use of whole tissue human gene expression profiling as an assay readout. Advancement in the use of human induced pluripotent stem cells may circumvent these issues (Muller et al., 2018). Likewise, HSEs containing Langerhans cells have been developed (Ouwehand et al., 2012, 2011b) and have been shown to respond to chemical stimulation (Bock et al., 2018; Ouwehand et al., 2011a). Combining all these cell types into a reproducible HSE that will respond to chemical insult in a standardized manner and that can be translated to industry will be challenging, although not impossible.

In conclusion, the expression of a seven-gene panel in HSE, based on immortalized KCs, in combination with multivariate statistical approaches shows enhanced confidence in the discrimination of skin irritants from nonirritants. This reproducible human in vitro assay offers potential in high-throughput compound assessment, but further testing of a larger chemical set is required to fully evaluate its predictive power.

## Materials and Methods

All reagents were purchased from Sigma-Aldrich (Gillingham, United Kingdom) and used as per the manufacturers’ instructions unless otherwise stated.

### Cell culture

TERT-immortalized human skin KCs (N/TERT-1; from Prof. Rheinwald) (Dickson et al., 2000) were cultured at low density in KC serum-free media (Thermo Fisher Scientific, Waltham, MA) supplemented with 25 μg/ml bovine pituitary extract, 0.2 ng/ml EGF, and 0.3 mM calcium chloride (total calcium ion concentration, 0.4 mM). Human dermal fibroblasts (HDFs) were isolated from skin biopsies obtained from the breast tissue of patients undergoing surgery with written, informed consent (ethical approval 09/H1308/66). Biopsies were incubated in 0.1% (w/v) trypsin solution supplemented with 100 IU/ml penicillin, 100 μg/ml streptomycin, and 0.625 mg/ml amphotericin B overnight at 4 °C. After enzymatic digestion, HDFs were isolated from the connective tissue by fine mincing followed by treatment with 0.25% (w/v) collagenase for 5 hours at 37 °C then collected by centrifugation and cultured in DMEM supplemented with 10% v/v fetal bovine serum, 100 IU/ml penicillin, 100 μg/ml streptomycin, and 2 mM L-glutamine. Cells were incubated at 37 °C in a 5% carbon dioxide humidified incubator, medium changed every 3 days, passaged when 80% confluent, and used up to passage 5.

### Generation of tissue-engineered HSEs

HSEs were constructed using N/TERT-1 as previously described (Dickson et al., 2000). In brief, rat tail collagen (5 mg/ml) was combined with 8% (v/v) fetal bovine serum, DMEM (×10), 2 mM L-glutamine, and reconstitution buffer (2.2% sodium bicarbonate, 4.8% HEPES, 0.25% sodium hydroxide in distilled H_2_O) and the pH adjusted to 7.4 with 2 M sodium hydroxide. HDFs (1.5 × 10^5^ per model) were added to the collagen mixture before transferring into 12-well translucent transwell inserts with 0.4 μm pores (Millipore, Burlington, MA) and allowed to set in a humidified atmosphere at 37 °C. Once set, 5 ml HDF culture media was added to the well and 500 μl onto the surface of the collagen and incubated for 24 hours at 37 °C. Next, 2.5 × 10^5^ N/TERT-1 cells were seeded on the collagen surface and cultured submerged in medium for 2 days, after which HSEs were raised to an air-to-liquid interface and further cultured for 14 days, changing the medium every other day.

### Ex vivo skin and HSE stimulation with chemical compounds

Skin explants from the breast tissue of independent donors were received from the theater in transport medium (DMEM with antibiotics), prepared by removing subdermal tissue, washed in PBS, and then used immediately in experiments. A 10-mm punch biopsy was taken and placed into a 0.4-μm pore transwell insert, and HDF culture media was added to the well underneath. A total of 50 μl of chemical compound was added to the surface of the ex vivo skin or HSE and incubated for 24 hours at 37 °C. Chemicals tested were LA (5% v/v), MP (methyl-4-hydroxybenzoate, 0.2% w/v), Co-DEA (2% v/v), Co-MEA (2% v/v), CAP (0.1% w/v), and CA (3% v/v). Chemicals were made up in H_2_O, which was also used as a vehicle CON. A skin biopsy from a different donor was used in each experiment, and each experiment was performed at least three times.

### LDH release assay

Cell damage was analyzed by measuring the release of LDH into the culture medium using a CytoTox96 enzyme assay kit as described in the manufacturer’s instructions (Promega, Madison, WI) as previously described (Jennings et al., 2016). Disruption of the epithelium using 5% v/v SDS was used as a positive CON.

### TEER

Tissue integrity was assessed by measuring TEER using an EVOM^2^ voltmeter (World Precicion Instruments, Madison, WI) at three locations per model, and the average of these values was calculated. A blank resistance measurement of the insert plus PBS was also measured for preconditioning. The following equation was used to measure TEER as previously described (Buchert et al., 2012): TEER Reported = Resistance (Ω) × Effective Membrane (cm^2^).

### RNA isolation

Skin and HSEs were washed with PBS before incubation with 15 U dispase for 2 hours at 37 ^o^C with frequent gentle mixing. The enzymatic reaction was stopped with PBS before centrifugation at 8,000*g* for 3 minutes, followed by further washes with PBS. Tri-reagent (Thermo Fisher Scientific) (300 μl) was added to the cell pellet and samples centrifuged at 8,000*g* for 3 minutes. The RNA-containing supernatant was removed, and the RNA was purified using RNeasy (Qiagen, Hilden, Germany), according to the manufacturer’s instruction. Total RNA (100 ng) was reverse transcribed using a high-capacity cDNA reverse transcription kit (Thermo Fisher Scientific), according to the manufacturer’s instructions.

### qPCR

qPCR was performed using TaqMan gene expression assays as follows: 0.5 μl cDNA was amplified using 5 μl master-mixture, 3.5 μl nuclease-free H_2_O, and 0.5 μl TaqMan gene probe (FAM); 0.5 μl β2-microglobulin (VIC) was used as a reference CON (Thermo Fisher Scientific). Reactions were performed using thermal cycles of 50 °C (2 minutes) and 95 °C (10 minutes), then 40 cycles of 15 seconds at 95 °C, followed by 1 minute at 60 °C. The threshold cycle was normalized against the reference gene and then fold-changes in expression relative to the H_2_O-treated CON group were calculated using the formula 2^**-ΔΔ**Ct^ (Livak and Schmittgen, 2001).

### Histological analysis

Skin and HSEs were fixed with 10% v/v neutral-buffered formalin; alcohol-processed, paraffin wax–embedded, 6-μm sections cut using a microtome; and sections stained with H&E. Slides were mounted with distyrene-polystrene xylene and imaged by light microscopy.

### Data analysis

All data are presented as mean ± SD unless otherwise stated, with all experimental repeats clearly stated. Data sets were checked for normality using the Shapiro-Wilk test. Parametric data was analyzed by ordinary one-way ANOVA with Dunnett’s multiple post-hoc test when comparing treatments to H_2_O vehicle-treated CONs or Tukey’s post-hoc test for multiple group comparisons, and differences were considered significant when *P* < 0.05. Statistical analysis was performed using GraphPad prism version 9.0 (GraphPad Software, San Diego, CA). Gene expression analysis was subjected to unsupervised hierarchical gene cluster analysis, and heatmap generation and PCA were conducted using Clustvis web tool (biit.cs.ut.ee/clustvis/). LDA was accomplished using RStudio (rstudio.com) along with R-package (https://cran.r-project.org/web/packages/MASS/index.html). A Github repository containing data analysis is available (Sting_predict).

### Data availability statement

No datasets were generated or analyzed during this study.

## ORCIDs

Amy L. Harding: https://orcid.org/0000-0003-3570-9338

Craig Murdoch: https://orcid.org/0000-0001-9724-122X

Simon Danby: https://orcid.org/0000-0001-7363-140X

Md Zobaer Hasan: https://orcid.org/0000-0001-7174-4443

Hirofumi Nakanishi: https://orcid.org/0000-0003-1353-0599

Tetsuo Furuno: https://orcid.org/0000-0003-3631-8910

Sirwan Hadad: https://orcid.org/0000-0002-9190-021X

Robert Turner: https://orcid.org/0000-0002-1353-1404

Helen E. Colley: https://orcid.org/0000-0003-0053-7468

## Author Contributions

Conceptualization: MZH, HEC, CM, SD; Formal Analysis: ALH, RT; Investigation: ALH, RT; Methodology: ALH, HEC, SD, CM; Project Administration: HEC, MZH, TF, HN; Resources: SH; Software: RT; Supervision: HEC; Visualization: ALH; Writing - Original Draft Preparation: ALH, CM, HEC; Writing - Review and Editing: ALH, CM, HEC, SD, MZH
